# Prevalence of Hepatitis B and C Among Prison Inmates in India: A Systematic Review and Meta-Analysis

**DOI:** 10.7759/cureus.19672

**Published:** 2021-11-17

**Authors:** Ajeet S Bhadoria, Kanchan B Gawande, Chandan K Kedarisetty, Bharat B Rewari, Vineet K Pathak, Pragya Pandey, Rohit Gupta

**Affiliations:** 1 Community and Family Medicine, All India Institute of Medical Sciences, Rishikesh, IND; 2 Centre for Community Medicine, All India Institute of Medical Sciences, New Delhi, IND; 3 Hepatology, Sri Ramachandra Institute of Higher Education and Research, Chennai, IND; 4 Epidemiology and Public Health, World Health Organization, South-East Asia Regional Office, New Delhi, IND; 5 Community and Family Medicine, All India Institute of Medical Sciences, Raipur, IND; 6 Gastroenterology and Hepatology, All India Institute of Medical Sciences, Rishikesh, IND

**Keywords:** india, prison, hepatitis c, hepatitis b, prevalence, meta-analysis

## Abstract

Viral hepatitis is still considered a major cause of the burden of disease in India. It is the most common cause of cirrhosis and liver cancer. Prisoners are one of the groups at most risk for hepatitis. This study aimed to estimate the pooled estimates of the prevalence of hepatitis B and C among prisoners in India.

The study followed the Preferred Reporting Items for Systematic Reviews and Meta-Analyses (PRISMA) guidelines for study selection. The extensive search was done through databases of PubMed, Embase, and Google Scholar. All cross-sectional studies conducted to find the prevalence of hepatitis B and C among prison inmates in India published till June 2020 were screened and included in this meta-analysis. The analysis was conducted using the random-effects model. The heterogeneity was estimated using the I2 indicator. After extracting the required data, the meta-analysis was performed using the software Stata, version 12 (StataCorp LLC, College Station, Texas). The study is registered in the International Prospective Register of Systematic Reviews (PROSPERO; registration no: CRD42020185137).

Out of a total of 970 articles searched through the database of PubMed, Embase, and Google Scholar, five studies that met the inclusion criteria were included and analyzed. Hepatitis B and C prevalence were given in four studies each. The results showed that the overall prevalence of hepatitis B and C in prisoners was 8% (95% CI: 4-12) and 7% (95% CI: 1-13). The studies show high heterogeneity with no evidence of publication bias. The prevalence of hepatitis B and C among male prisoners was 4.48% (95% CI: 3.64%-5.32%) and 6.35% (95% CI: 5.48%-7.23%), respectively, while the prevalence among female prisoners was 1.53% (95% CI: 0.31-2.75) and 2.10% (95% CI: 0.28-3.93), respectively.

The study findings show a high prevalence of hepatitis B and C in prisoners, which is of particular concern. Appropriate and effective interventions to reduce the transmission of hepatitis B and C in prisons are essential.

## Introduction and background

Viral hepatitis is one of the common viral diseases that are present worldwide and its most important variants are hepatitis B and C [1]. Hepatitis is responsible for more than two million annual deaths worldwide because of its complications like liver cirrhosis, liver failure, and liver cancer [2]. The prevalence of hepatitis B is alarming globally, and it has been found that 5% of the global population is suffering from hepatitis B infection, which in absolute number comes to around 350 million people [3]. The rate of new infection annually is also high for hepatitis B; nearly 4 million people acquire the infection each year and the number of lives lost to the disease is nearly 1 million per year [4]. The geographical distribution of hepatitis B is also variable ranging from as little as 0.1%-0.5% to as high as 5%-20% in regions of Europe and eastern tropical countries [5]. One successful strategy for the prevention of hepatitis B is immunization against the same, which is reported the most cost-effective intervention too [6].

Hepatitis C also has a global prevalence of approximately three per 100 individuals, contributing to more than 170 million chronic cases globally. Along with hepatitis B, hepatitis C also contributes to chronic diseases like liver cirrhosis, cancer, and premature mortality due to complications [7]. As per the Global Hepatitis Report (2017), WHO estimates that 71 million persons were living with hepatitis C virus (HCV) infection in the world that requires life care to prevent complications of the disease [2]. In the general population, hepatitis C prevalence varies from 2% to as high as 18% in some regions [8].

In view of such a huge burden of hepatitis, the vulnerable populations are among the hard-hit section of the society because of many epidemiological parameters like poor nutrition, poor hygiene, high-risk behavior, poor socioeconomic status, and poor awareness about the preventive strategies. One of the groups that are at the highest risk for hepatitis is prisoners in jail [9]. Various studies have reported a higher prevalence of hepatitis among prisoners, which is as high as nine times as compared to the general population [10,11].

In prisons, owing to their cohabitation in a closed environment for a long period of time and exposure to various risk factors makes them more vulnerable to the infection. Thus, prisoners are susceptible to infectious diseases and, after release from prison, they might act as a bridge population to spread the disease in the community [8]. People referred to correctional centers often experience drug injection, needle sharing, and sexual risk behavior, which all contribute to a high risk of transmission of many blood-borne infections.

Numerous studies have been done on hepatitis B surface antigen (HBsAg) and HCV prevalence among the general population in India. But unlike other countries, there is a scarcity of published data on hepatitis B virus (HBV) and HCV infections among prisoners in India despite its tremendous importance in public health formulation compared to the general population.

Since one of the neglected groups of individuals who are at risk for acquiring the infection are prisoners and viral hepatitis is one of the diseases of public health importance, this study was conducted to obtain an estimate of the prevalence of viral hepatitis among prisoners in India.

## Review

Methodology

Study Selection

**Figure 1:**
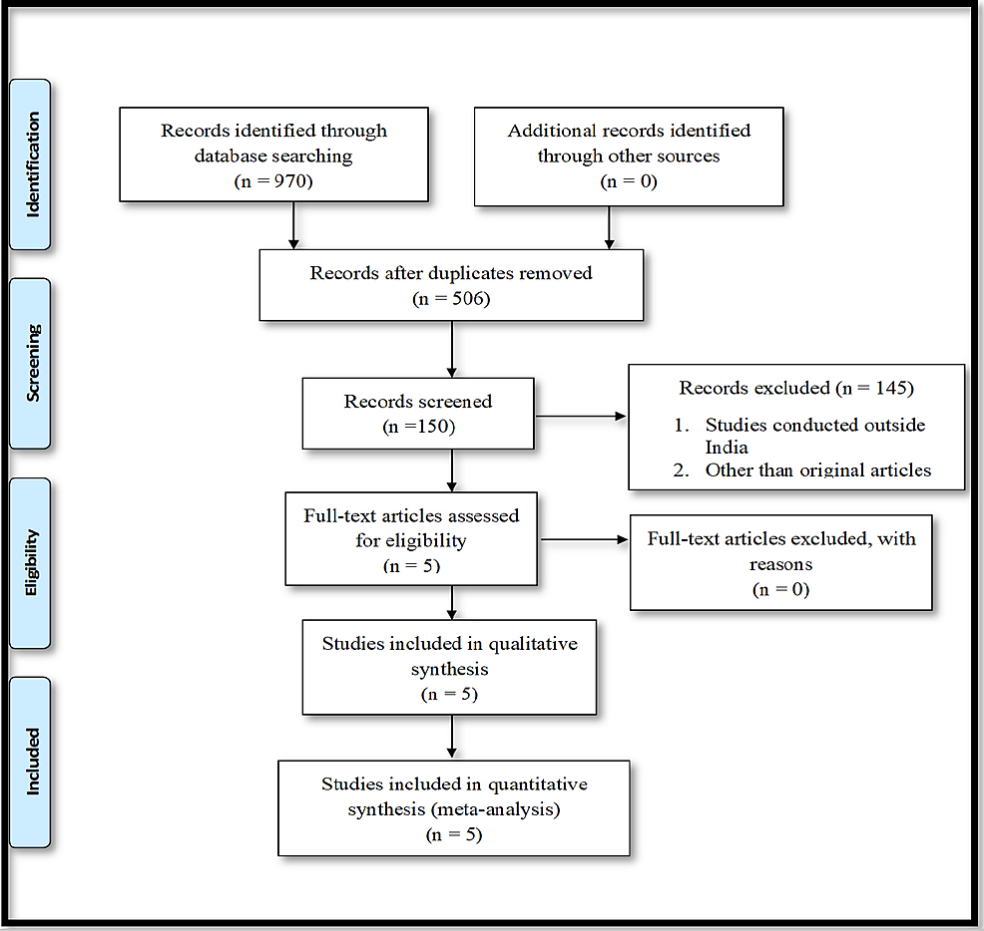
PRISMA flow diagram for study selection for meta-analysis. PRISMA, Preferred Reporting Items for Systematic Reviews and Meta-Analyses.

We conducted this systematic review and meta-analysis to assess the pooled prevalence of hepatitis B and C infection among prisoners in India. Data were obtained using different databases such as PubMed, Cochrane Library, and Embase. The Preferred Reporting Items for Systematic Reviews and Meta-Analyses (PRISMA) [12] guidelines were followed for study selection. Articles providing data on hepatitis B and C prevalence among prison inmates in India are included in this study. After a detailed assessment, a total of five articles were included in the analysis. A total of 970 articles were found on the databases, 506 of which were duplicated and removed through title screening. After the screening of all the retrieved records, 145 articles were removed. A total of five full-text studies were assessed for eligibility and finally included in the meta-analysis (Figure 1).

Search Strategy

The following keywords were searched: ("Hepatitis B" [Mesh]) AND ("prisons" [Mesh: No exp]) AND ("prevalence" [Mesh]), ("Hepatitis C" [Mesh]) AND ("prisons" [Mesh: No exp]) AND ("prevalence" [Mesh]), as well as their derivatives. The obtained references both primary and secondary were also evaluated to increase the probability of finding other literature sources that can be included in the study.

Inclusion Criteria

All observational cross-sectional studies published till June 2020 in the English language and reporting the prevalence of hepatitis B and C among prisoners in India were included. The outcome measure of every study should be the number of participants (prison inmates) infected with hepatitis B and C.

Data Extraction

The data used in the present study were extracted from the previous studies through the use of a Microsoft Excel sheet (Microsoft Corp., Redmond, WA). For all included studies, we have recorded publication year, author name, study design, sample size, study area, number of hepatitis b and c positive prisoners, and exposure characteristics like i.e. injection drug users among participants and participants who had unprotected sex with commercial sex workers (CSWs).

Quality Assessment

The risk of bias/quality assessment of all the articles included was done by two authors (ASB and KBG). Newcastle-Ottawa assessment checklist [13] for observational studies was applied for assessing the quality of each study considered in this research. The tool has three sections: the first section (methodological assessment/selection), the second section (comparability evaluation), and the third section (outcome). All the included articles were assessed through the tool. There was a joint discussion between the authors for uncertainty.

Statistical Analysis

After extracting the required data, data were entered into Microsoft Excel and transferred to STATA software, version 12 (StataCorp LLC, College Station, TX) for the meta-analysis. In this study, the pooled weighted average was used in the evaluations. The random-effects model was used to determine the hepatitis B and C prevalence considering heterogeneity among studies. To examine heterogeneity, its quantity was estimated using the I2 indicator (I2 is the percentage of total variation across studies due to heterogeneity rather than chance). The publication bias was checked using Egger’s linear regression method and a p-value of less than 0.05 was used to declare its statistical significance.

Results

Characteristics of Included Studies

Of the five articles [14-18] that were evaluated in our study, three were from New Delhi [14-16], one was from Tamil Nadu [17], and one was from Uttar Pradesh [18]. All studies were conducted using a cross-sectional study design. Only one study was conducted and published before the year 2000 [14] while the remaining four were published after the year 2000 [15-18].

The sample size in these studies varied from 50 to 1,611 participants. Nearly 2,782 participants were screened for hepatitis B and 3,291 screened for hepatitis C. The prevalence of hepatitis B among the participants was 5% (145/2,782), while the prevalence of hepatitis C was 6% (206/3,291). Three studies [14,17,18] reported the prevalence of injection drug users among prisoners. Nearly 10% (313/3,241) of prisoners reported as injection drug users range from a minimum of 1.79% to a maximum of 20%. Three studies reported the prevalence of prisoners with a history of unprotected sexual intercourse with CSWs [14,17,18] and 40.92% (1,798/4,393) of prisoners reported unprotected sexual intercourse with CSWs ranging from a minimum of 34.58% to a maximum of 81% (Table 1). Also, on the application of the Newcastle-Ottawa quality assessment checklist [13], the final score of selected studies is mentioned (Table 2).

**Table 1:** General characteristics of individual included studies (N = 5). CSW, commercial sex workers.

Sr. No.	Authors	Study year	Year of publication	Study design	Study area	Study population (N)	No. of prisoners positive for hepatitis B	No. of prisoners positive for hepatitis C	Injection drug users among prisoners (%)	Prisoners with a history of unprotected sex with CSW (%)
1	Singh et al. [14]	1999	1999	Cross-sectional study	New Delhi	249	30	12	3.33	34.58
2	Kar et al. [15]	2000	2000	Cross-sectional study	New Delhi	50	17	8	-	-
3	Rana et al. [16]	2015	2015	Cross-sectional study	New Delhi	1,102	30	-	-	-
4	Ramamoorthy et al. [17]	2016	2016	Cross-sectional study	Chennai	1,381	68	18	20	81
5	Tyagi et al. [18]	2018	2018	Cross-sectional study	Uttar Pradesh	1,611	-	168	1.79	36.9

**Table 2:** Total score of individual studies on the application of the Newcastle-Ottawa quality assessment checklist.

Criteria	Sub-criterion	Studies (N = 5)				
		Singh et al. [14]	Kar et al. [15]	Rana et al. [16]	Ramamoorthy et al. [17]	Tyagi et al. [18]
Selection	Representativeness of the sample	1	1	1	1	1
	Sample size	1	0	1	1	1
	Non-respondent	0	0	1	0	0
	Ascertainment of the exposure (risk factor)	0	0	0	0	0
Comparability	Confounding factors are controlled	1	1	1	1	1
Outcome	Assessment of the outcome	2	2	2	2	2
	Statistical test	1	1	1	1	1
Total score		6	5	7	6	6

Prevalence of Hepatitis B

**Figure 2:**
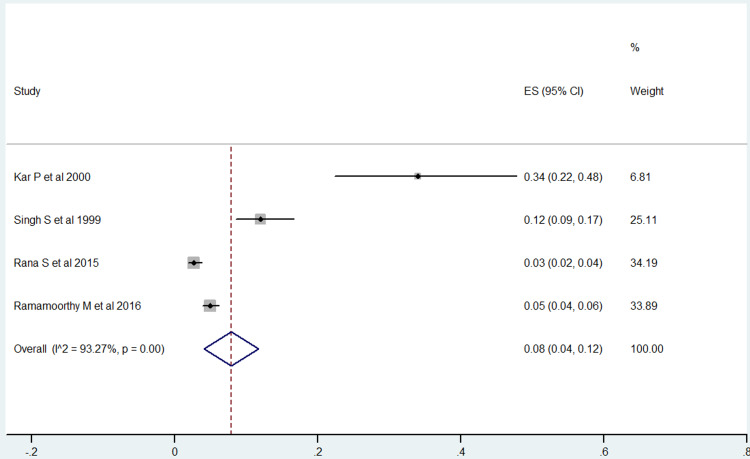
The pooled prevalence of hepatitis B among prison inmates in India. ES, effect size.

**Figure 3:**
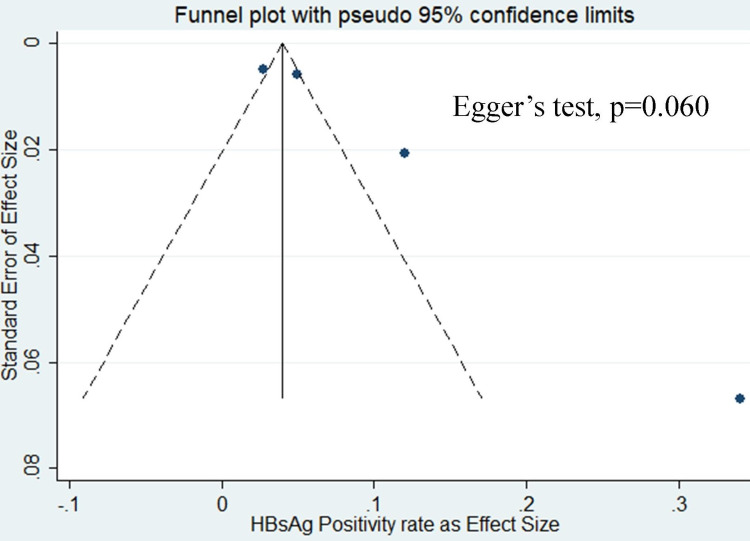
Funnel plot for publication bias among studies reported with hepatitis B prevalence among prison inmates in India. HBsAg, hepatitis B surface antigen.

From the five studies included in our analysis, four of them reported the prevalence of hepatitis B in prisons (Figure 2) [14-17]. Analysis of these studies showed that the pooled prevalence of hepatitis B in prisoners was 8% (95% CI: 4-12). A heterogeneity test showed evidence of high heterogeneity (I2 = 93.2% and p-value = <0.001), whereas Egger’s tests showed no statistical evidence of publication bias (p-value = 0.06) (Figure 3). The results also indicated that among the studies conducted in different states, the prevalence of hepatitis B for three studies reported from New Delhi was 5.49% (95% CI: 4.30-6.68), while as for the single study reported from Chennai, Tamil Nadu, hepatitis B prevalence was 4.92% (95% CI: 3.78-6.06). As for male and female, hepatitis B prevalence was higher in males i.e. 4.48% (95% CI: 3.64%-5.32%) than females i.e. 1.53% (95% CI: 0.31-2.75).

Prevalence of Hepatitis C

**Figure 4:**
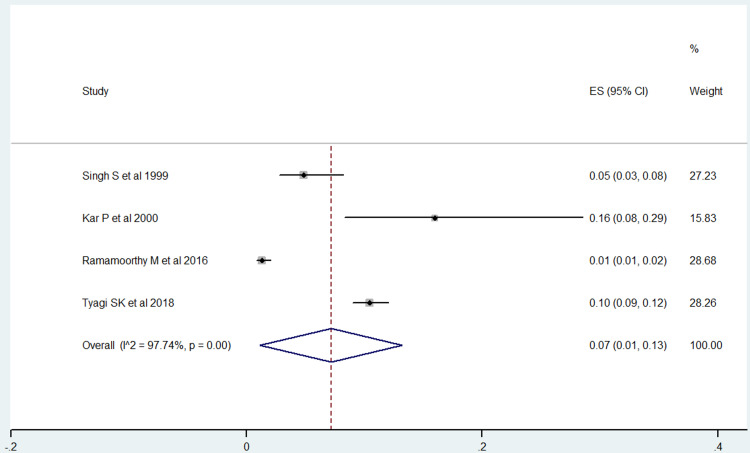
The pooled prevalence of hepatitis C among prison inmates in India. ES, effect size.

**Figure 5:**
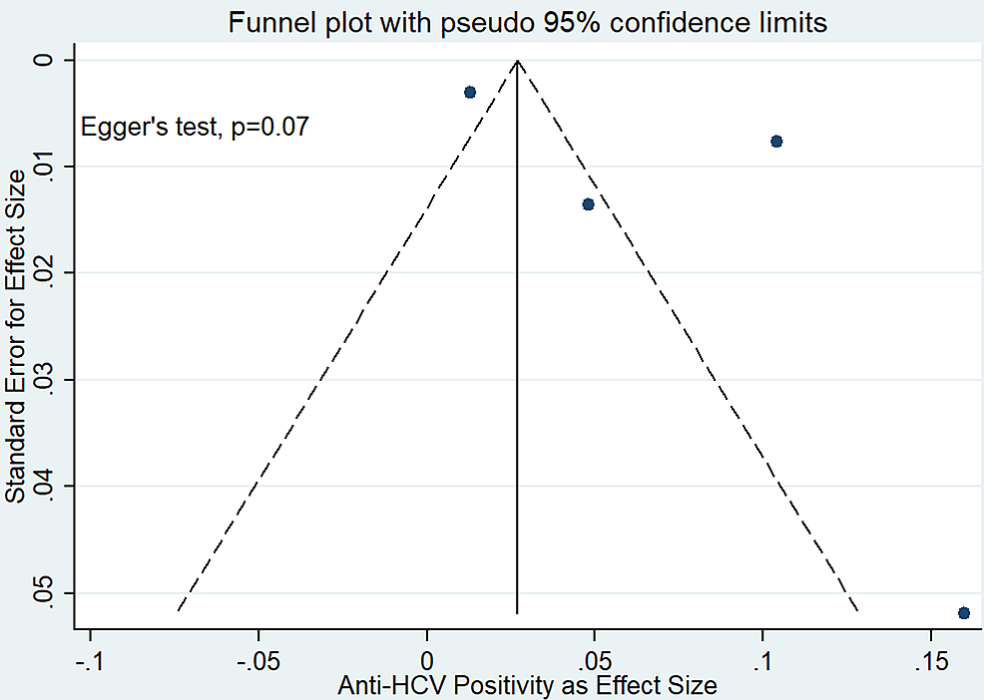
Funnel plot for publication bias among studies reported with hepatitis C prevalence among prison inmates in India. HCV, hepatitis C virus.

Among the five studies included in our analysis, four studies reported on the prevalence of hepatitis C among prison inmates (Figure 4) [14,15,17,18]. Analysis of these studies indicated that the pooled prevalence of hepatitis C among prisoners in India was 7% (95% CI: 1-13). The heterogeneity test showed evidence of high heterogeneity (97.74% and p-value ≤ 0.001). However, there was a non-significant publication bias (Egger’s test, p-value = 0.347) (Figure 5).

Among the studies conducted in different states, the prevalence of hepatitis C for two studies reported from New Delhi was 6.68% (95% CI: 3.85-9.52), while as for the single studies reported from Tamil Nadu and Uttar Pradesh, the hepatitis C prevalence was 1.30% (95% CI: 0.70-1.90) and 10.42% (95% CI: 8.93-11.92), respectively. As for male and female, hepatitis C prevalence was higher in males i.e. 6.35% (95% CI: 5.48%-7.23%) than females i.e. 2.10% (95% CI: 0.28-3.93).

Discussion

Prisoners are considered as most marginalized members of society. They have risky behavioral patterns, violent tendencies, deviant sexual behavior, substance abuse, and negligence in terms of health services. This group is facing double punishment in terms of incarceration followed by acquired illnesses. In certain cases, the latter one has the potential to get inflicted on the family of prisoners on their release [19,20]. The common use of injection materials, tattooing, and other circumstances that result in blood contact increase the risk of infections. One of the main health-related concerns among prisoners is blood-borne infections, including hepatitis B and C [21,22].

This study aimed to estimate the pooled prevalence of hepatitis B and C among prisoners in India. In the present study, five studies were reviewed to find the prevalence of hepatitis B and C. In the final meta-analysis, four articles each were included to estimate the prevalence of hepatitis B and C. The heterogeneity of the prevalence rates for hepatitis B and C was 93.2% and 97.74%, respectively, indicating high heterogeneity. The random-effects model, therefore, was used for further analysis.

The pooled prevalence of hepatitis B and C in the present study was 8% and 7%, respectively. Another meta-analysis was conducted to estimate the prevalence of hepatitis b and c among prisoners worldwide. This meta-analysis included 31 studies conducted in 17 countries of five WHO regions to estimate the prevalence of hepatitis B and 38 studies from 20 countries of six WHO regions to estimate the prevalence of hepatitis C from the year 2005 to 2015. However, this meta-analysis did not mention any study conducted in India. The meta-analysis revealed pooled prevalence of hepatitis B and C worldwide as 5.17% and 13.22%, respectively [23]. This meta-analysis also found the highest prevalence of hepatitis C among prisoners was in the Southeast Asia region, at 24.26% [23]. A study conducted in Kahramanmaras, Turkey in 2016 found the prevalence of hepatitis B and C among prisoners as 2.6% and 17.7%, respectively [24]. A study conducted by Shefat in 2018 at Porto Velho, Rondonia, Brazil found the prevalence of hepatitis B and C among prisoners as 1.4% and 0.9%, respectively [25]. Some of the recent studies conducted in the year 2019 among prisoners reported the prevalence of hepatitis B and C as 1.9% and 17.0% at Stockholm County, Sweden [26] and 4.7% and 0.5% in Turkey [27].

The gender analysis showed that the overall prevalence of hepatitis B and C in men was 4.48% and 6.35%, which was comparable to the result shown by a worldwide meta-analysis study, which reported prevalence among men as 6.7% and 9.33%, respectively [23]. Although for hepatitis B, this prevalence is much higher when compared to studies conducted in Brazil and Sweden, which were 1.4% and 2.1 %, respectively [25,26], while in a study conducted in Sweden, the prevalence of hepatitis C among men is reported as high as 16%. The results showed that the overall prevalence of hepatitis B and C among female prisoners was 1.53% and 2.10%, which was much less when compared to a worldwide meta-analysis, which was 4.34% and 6.25%, respectively [23]. A study conducted in Brazil reported no female prisoner infected with hepatitis B and C [25] while 33.3% of female prisoners reported being infected with hepatitis C in Sweden [26].

As per the findings of this meta-analysis, it could be argued that the prevalence of hepatitis among males is greater when compared with female prisoners. This could reflect greater risk behavior and drug injection among men. Drug abuse is a global concern, with about 5% of the world population exposed to drug abuse in one or another form [28]. Injectable drug abuse is harmful and a route to many types of blood-borne infections. Also, spending so much time in prison increased their risk of unprotected intercourse, engaging with men who have sex with men (MSM), etc. [29]. The present study also reported a much higher prevalence of unprotected sexual intercourse with CSWs (40.92%) and injection drug use (9.65%) among prisoners. Considering this, the risk among prisoners is much high as they live in a closed environment and use drug injection with common needles, which could exacerbate the transmission of blood-borne diseases. These findings affirm the greater need for training in this group for the prevention and management strategies of the disease.

## Conclusions

The study findings showed a high prevalence of hepatitis B and C among prisoners, which is of particular concern. Risk behaviors such as injection drug use are common in prisoners. The population of the prisoners harbors diseases that are determined both by the environment from which they come and in prison in which they live. They form a cohort inside the prison and if any index case is developed among them, the chances of spread can be tremendous. The prisoners along with the common man have an equal right to good care inside the prison. Hence, regular screening for early detection and adequate health education should be given to prisoners in health planning and prevention to prevent the further spread of the disease in prisons, to their family members, and in the community. Also, this meta-analysis shows there is a need for more studies on the health indicator of the prisoners who are neglected and the same time high-risk groups of the society.
